# Long-Lasting, Kin-Directed Female Interactions in a Spatially Structured Wild Boar Social Network

**DOI:** 10.1371/journal.pone.0099875

**Published:** 2014-06-11

**Authors:** Tomasz Podgórski, David Lusseau, Massimo Scandura, Leif Sönnichsen, Bogumiła Jędrzejewska

**Affiliations:** 1 Mammal Research Institute, Polish Academy of Sciences, Białowieża, Poland; 2 Institute of Biological and Environmental Sciences, University of Aberdeen, Aberdeen, United Kingdom; 3 Department of Science for Nature and Environmental Resources, University of Sassari, Sassari, Italy; Institute of Biology, University Leipzig, Germany

## Abstract

Individuals can increase inclusive fitness benefits through a complex network of social interactions directed towards kin. Preferential relationships with relatives lead to the emergence of kin structures in the social system. Cohesive social groups of related individuals and female philopatry of wild boar create conditions for cooperation through kin selection and make the species a good biological model for studying kin structures. Yet, the role of kinship in shaping the social structure of wild boar populations is still poorly understood. In the present study, we investigated spatio-temporal patterns of associations and the social network structure of the wild boar *Sus scrofa* population in Białowieża National Park, Poland, which offered a unique opportunity to understand wild boar social interactions away from anthropogenic factors. We used a combination of telemetry data and genetic information to examine the impact of kinship on network cohesion and the strength of social bonds. Relatedness and spatial proximity between individuals were positively related to the strength of social bond. Consequently, the social network was spatially and genetically structured with well-defined and cohesive social units. However, spatial proximity between individuals could not entirely explain the association patterns and network structure. Genuine, kin-targeted, and temporarily stable relationships of females extended beyond spatial proximity between individuals while males interactions were short-lived and not shaped by relatedness. The findings of this study confirm the matrilineal nature of wild boar social structure and show how social preferences of individuals translate into an emergent socio-genetic population structure.

## Introduction

A variety of social systems arise from the attempts individuals make to directly or indirectly maximise their fitness. Cooperative interactions among animals, maintained by mutualism and behavioural reciprocity, can provide individuals with assets vital for survival and reproduction [1], [2]. Animals can also obtain indirect fitness benefits by interacting with related individuals [3]. Individuals can increase inclusive fitness by kin-directed cooperative behaviours such as sharing information about resources, predation avoidance, cooperative foraging and breeding [4]–[6]. Such benefits promote philopatry, leading to the evolution of kin-based social structures. Indeed, kin-based social systems are common across a variety of mammalian species, e.g. polar bear *Ursus maritimus* [7], sperm whale *Physeter macrocephalus* [8], gray mouse lemur *Microcebus murinus* [9], woodchuck *Marmota monax* [10], and Florida black bears *Ursus americanus floridanus* [11]. In some species, however, relatedness is not the main determinant of social structure e.g. raccoon *Procyon lotor* [12], southern flying squirrels *Glaucomys volans* [13].

Matrilineality (females associated by pedigree through female ancestors) is a widespread type of social organisation among suids, e.g. babirusa *Babyrousa babyrussa* [14], warthog *Phacochoerus africanus* [15], and desert warthog *Phacochoerus aethiopicus* [16]. Wild boar social structure is centred around family groups of adult female (s) with offspring [17], [18]. Commonly, a few families merge to form matrilineal and multigenerational social units, which occasionally break, reform, or exchange individuals [19], [21]. Cohesive social groups of related individuals [20], [21] and female philopatry [22] of wild boar create conditions for cooperation through kin selection and make the species a good biological model for studying the effects of kinship on social behaviour. Yet, these effects are poorly understood in wild boar populations. Iacolina et al. [23] found low levels of intra-group relatedness, no correlation between genetic and spatial distance among adults, and frequent associations of unrelated females. The apparently weak kin-structure in this study was attributed to high human-caused mortality altering social structure and wolf *Canis lupus* predation pressure stimulating unrelated individuals (hunting survivors) to associate. In contrast, Poteaux et al. [21] showed that females in spatial proximity were more related than expected by chance, thus providing some evidence for a kin-based, matrilineal population structure. Both studies were conducted in heavily hunted populations with potentially strongly perturbed social structures. In this study, we investigated a wild boar population with minimal exposure to anthropogenic factors, a situation rarely found in Europe. Additionally, analyses relating kinship and social behaviour may be biased due to inaccuracy of inferring relatedness with microsatellites markers [24], [25]. Therefore, more studies are needed to resolve conflicting patterns observed in wild boar populations.

Social structure emerges from the non-random distribution, grouping, and ranging patterns of individuals in a population [26]. Identifying occurrence, distribution, and composition of social groups helps to reveal individual association preferences and is essential to determine a population’s social structure [27]. Dyadic interactions are the basic elements upon which social structure is built. They can be approximated by recording situations in which interactions might potentially occur, such as dyadic spatial proximity (association) [27]. Therefore, measuring the time two animals spend together using association indices offers a convenient, yet qualitatively simplified, substitute to estimating actual interactions and, consequently social relationships [28]–[30]. Analysing the rate at which associations between individuals change over time can help characterise the temporal aspect of social structure dynamics [31], [32]. Describing the structural properties of a social system requires accounting for the spatial and temporal organisation of the individuals’ association patterns and a network approach offers a powerful tool to explore such complex, dynamic systems [33], [34]. Our study is among the few, and first in wild boar, to address the relationship between social behaviour, space use and kinship under the network perspective [12], [35]–[37].

In this study we identified the community structure of the wild boar population, evaluated the influence of spatial, genetic, and temporal effects on the emergent social structure, and explored the relationship between relatedness and the strength of social bonds. Assuming matrilineal social structure in wild boar, we predicted that: a) associations of females will be temporarily stable and long-lasting, b) there will be a positive correlation between relatedness and the strength of social bonds among females, c) individuals, particularly females, of the same social groups will be more related to each other than the wider population background owing to cross-generational site fidelity.

## Methods

### Ethical Statement

The trapping of wild boar was carried out with the permission of the Ministry of Environment of the Republic of Poland (decision no. DLgł-6713/12/08/ab). The Director of the Białowieża National Park approved field work, including trapping and telemetry, in the area of Białowieża National Park (permit issued on 08.04.2008). The research and handling protocol (see below for detailed trapping procedure) was reviewed and approved by the Local Ethical Commission for Experiments on Animals in Białystok, Poland (resolution no. 19/2008). The wild boar population in the study area did not routinely receive any veterinary treatment (e.g. vaccinations). However, each trapping event was supervised by an appointed veterinarian in case medical intervention was needed.

### Study Area

The study was conducted in Białowieża Primeval Forest (BPF), a continuous forest complex of 1450 km^2^ (52°30′–53°00′N, 23°30′–24°15′E) that straddles the Polish-Belarusian border. The BPF is the last remnant of European temperate lowland forest and is unique among other European woodlands due to its high proportion of natural stands, old-growths, and the outstanding diversity of flora and fauna [38]. The native wild boar population is largely shaped by natural factors, such as mean annual temperature, acorn crop, winter severity, and wolf predation [38]. Most of the Polish part of the BPF (83%) is managed by the State Forestry, while the rest comprises the Białowieża National Park (BNP). Within the BNP, hunting and logging is prohibited, and tourist access is restricted. Within the commercial part of the BPF, limited hunting from fixed locations is only permitted at designated sites. In 2008–2011, the density of wild boar in the study area averaged 4 ind./km^2^ (unpublished data of the Mammal Research Institute, Polish Academy of Sciences). Within the managed part of the BPF, average hunting harvest was 0.9 ind./km^2^ (Regional Directorate of the State Forests, Białystok). Genetic sampling covered the entire BPF complex, while trapping and telemetry took place in the study area located in the centre of the Polish part of the BPF. Two-thirds of the study area (including all trapping locations) was within the borders of the BNP. The remaining part of the study area, where some animals were located temporarily, was within the commercial section of the BPF.

### Data Collection

#### Wild boar trapping and telemetry

Sex and age determination, genetic sampling, and tagging/collaring of wild boars were carried out during live-trapping conducted in 2007–2010. Two methods were used to capture wild boar: large drop-net traps [39] and cage traps (1.5×1×2 m), both baited with maize. During a trapping event, the drop-net traps were surveyed with a wireless monitoring system from a distance of 200–300 meters. The net was released remotely by the researchers when a group of wild boar centred under the net. Self-triggered cage traps were equipped with an alarm system sending information about trap closure via GSM network, which allowed for the quick release of captured animals. The number of animals trapped at once varied from 1 to 15 with drop-net traps and from 1 to 3 with cage traps. A mixture of tiletamine and zolazepam – Zoletil (Virbac, Carros, France) and medetomidine – Domitor (Orion Pharma, Espoo, Finland) mixture (1∶0.025 ratio) was administered intramuscularly to anaesthetise captured wild boar [40]. Atipemazole hydrochloride – Antisedan (Orion Pharma, Espoo, Finland) was used as an antidote [40]. Animals weighing less than 30 kg were only immobilised with ketamine (0.2 ml/kg) – Bioketan (Vetoquinol Biowet, Gorzów Wlkp., Poland) and were handled without being fully anaesthetised. The drugs were administered while animals were in the traps and the doses were wild boar specific [40]. Captured animals were fitted with ear tag radio-transmitters (Advanced Telemetry Systems, Isanti, Minnesota, USA and Wagener Telemetrieanlagen, Cologne, Germany) or, on adults only, GPS collars (Vectronic Aerospace, Berlin, Germany). Radio ear-tags weighed approximately 40 g and GPS collars approximately 800 g which were 0.08% and 1.6% of the mean body weight of marked animals, respectively. We did not observe any adverse effects of trapping, tagging or collaring on wild boar behaviour or survival during the monitoring period. During all trapping events at least one person authorised to administer drugs was present. On average, handling of animals until full recovery did not last longer than 45 minutes.

Upon capture, the age of wild boar was determined with 2-month interval accuracy based on tooth eruption [41]. In the analyses, animals were assigned to their respective age classes during the tracking period; i.e. yearlings (from 6–8 to 16–18 months old), subadults (from 16–18 to 24–26 months) and adults (>26 months old). Sex was determined for all individuals except one yearling which was excluded from analyses investigating sex-related effects. A total of 106 wild boars were captured, including 6 re-captures (at least one year after the first capture): 6 adult, 5 subadult and 27 yearling males as well as 18 adult, 14 subadult and 35 yearling females. The proportion of individuals marked with telemetry transmitters out of all captured animals (i.e. mark rate) was 55%. Telemetry-marked animals included all wild boar captured alone and on average 60% of the group’s members, always including all the adults and subadults within captured groups.

The study area was surveyed 2–4 times per week, with equal intensity during the day and night, with an attempt to locate all marked animals within one day. The locations of individuals were determined on foot by recording at least 3 bearings for each triangulation using a 3-element Yagi antenna (Titley Scientific, Lawnton, Australia) and Yaesu FT-817 transceiver (Yaesu Musen Co., Tokyo, Japan). A vehicle was used to move about the study area. The location of an individual was calculated from a given set of bearings using the maximum likelihood estimator method [42] as implemented in the program LOAS (Ecological Software Solutions). The accuracy of triangulation was determined in the field by placing transmitters in known locations [43]. Our accuracy for the mean estimated error between known transmitter locations and those obtained from telemetry was 153±9.8 m (mean ± SE, *n* = 120). Wild boars were radio-followed for 8.9±0.5 (mean ± SE) months in 2008 and 7.3±0.5 months in 2009 and the mean (± SE) number of locations per individual per month was 7 (±0.3) and 6 (±0.3) in 2008 and 2009, respectively.

#### Sample collection and genetic analyses

For a total of 411 individuals from the BPF, including all animals used in this study, genomic DNA was extracted from tissue (*n* = 386) and hair (*n* = 25) samples. The majority of samples (*n* = 300) were obtained from animals that were hunted or found dead (220 in the Polish and 80 in the Belarusian parts of the BPF). The remaining 111 samples were collected from individuals captured in 2007–2010. Skin samples (an ear fragment of 5 mm in diameter) from captured and anaesthetised animals were obtained using a standard biopsy punch. The punched location area was treated with antibacterial topical spray Fatroximin (Fatro, Ozzano Emilia, Italy) to facilitate healing and reduce the risk of infection. Hair samples were obtained by plucking out 10 hairs with bulbs. Each individual was genotyped using one type of sample, i.e. hair or tissue. Animals were sampled and identified in the field when hunted, trapped or found so there was no risk of individual misidentification.

Genomic DNA was extracted using GenElute Mammalian Genomic DNA Miniprep kit (Sigma-Aldrich, St. Louis, Missouri) for tissue samples and Instagene Matrix (Bio-Rad, Hercules, California) for hair samples, and kept at −20°C. All individuals were genotyped with a panel of 16 polymorphic microsatellite loci (S090, SW72, S155, S026, S355, S215, SW951, SW857, SW24, SW122, IGF1, SW461, SW1492, SW2021, SW2496, SW2532), which had previously been successfully used to study relatedness and genetic variation in wild boar populations [23], [44]–[46]. Polymerase chain reaction (PCR) was performed in 10 µl reaction volume, containing 3 µl of DNA solution, 0.5 U of *Taq* DNA polymerase (Euroclone, Siziano, Italy), 1 U PCR buffer (Euroclone), 2.5 mM MgCl_2_, 100 µM of each deoxynucleotide triphosphate (dNTP), and 2 pmol of each primer. The forward primer of each pair was labelled with an ABI fluorescent dye (6-FAM, HEX, or NED; Applied Biosystems, Foster City, California). The amplification profile was set up with an initial step of denaturation at 95°C for 3 minutes, followed by 35 cycles of 92°C for 45 seconds, annealing temperature (52–65°C) for 45 seconds, and 72°C for 30 seconds. A further extension step of 72°C for 10 minutes concluded the reaction. PCR-amplified microsatellite alleles were sized using capillary electrophoresis in an ABI PRISM (Applied Biosystems) automatic sequencer at the BMR-Genomics (Padua, Italy). Peak Scanner software (Applied Biosystems) was used to visually inspect electropherograms for scoring alleles. Genotypes with ambiguous electropherograms were repeated.

The presence of scoring errors or null alleles in the dataset was evaluated using MICRO-CHECKER 2.2.3 [47], which detects signals of stuttering or large allele dropout, as well as an excess of homozygotes due to presence of null alleles. A correlation between amount of homozygotes and amount of missing data across individuals and loci is expected if a dataset is affected by allelic dropout due to low DNA concentration or poor sample quality [48]. We tested for such correlation using MICRODROP 1.01 [48].

Queller and Goodnight estimator of pairwise genetic relatedness [49] was obtained among all sampled individuals (*n* = 411) with GENALEX 6.4 [50] and subsequently used in all analyses restricted to animals for which telemetry data were available. Basic parameters of microsatellite polymorphism and genetic diversity were calculated using GENALEX 6.4 and FSTAT [51]. GENEPOP 4.0 [52] was used to test loci for departures from linkage equilibrium and the Hardy-Weinberg equilibrium (HWE) using the Markov chain method (parameters: 5000 dememorisation steps, 100 batches, 1000 iterations/batch). The significance level was adjusted for multiple testing across loci using the sequential Bonferroni correction [53].

### Data Analysis

#### Association analysis and social network structure

Association and network analysis was based on radio-telemetry data from 47 wild boar collected in 2008 and 2009. The two years were treated separately as the sets of marked animals in both years did not fully overlap. To determine pairwise associations we only used simultaneous locations of the dyads, i.e. two animals located within one hour interval. The mean (± SE) number of such simultaneous dyadic locations per month was 472 (±149) and 210 (±46) in 2008 and 2009, respectively. Two individuals were defined as being associated if they were located simultaneously (<1 hour) within a 350 m distance from each other. Although most recorded associations occurred at short distances (0–50 m: 52% of associations, 0–150 m: 75% of associations), we retained the 350-m threshold to include all potential associations taking into account radio-tracking error, i.e. situations when two animals were in fact together but their estimated locations could have been biased due to radio-tracking error. We commonly observed wild boar groups spread over such distances, especially when foraging or travelling. This threshold was more conservative than previously used (500 m in Iacolina et al. [23]).

The strength of dyadic associations was calculated using the half-weight index (HWI) [54], with a one day sampling period to mirror the actual sampling schedule. The HWI ranges between 0 (two individuals never located together) and 1 (two individuals always located together). The HWI estimates for each year were calculated using SOCPROG 2.4 [55] in MatLab 7.7.0 (The Mathworks Inc., Natick, Minnesota, USA). Two networks of 31 (year 2008) and 30 (2009) interconnected animals were constructed and visualised in NETDRAW [56]. To test whether the observed association patterns differed from random, the association data was randomly permuted 1000 times and mean HWI and its coefficient of variation (CV) were compared between real and randomised data sets [55], [57]. A significantly higher CV of real association indices compared to randomised data indicates the presence of long-term preferred companions in the population [55].

Population structuring was determined from association data by finding an optimal subdivision of the social network into a number of clusters (hereafter social units) using modularity matrix clustering [58], [59]. This method finds optimal network structure through an iterative process of dividing the network into a number of clusters from one to *n*, where *n* is the number of individuals forming the network. At each step, the number of edges (connections) within and between clusters is quantified by the modularity index *Q*. The most parsimonious division in the network (the one maximising *Q*) provides the most edges within clusters and the least between. Network structure analysis was performed in SOCPROG and visualised with NETDRAW.

#### Genetic and spatial effects on network structure

Spatial overlap between areas utilised by two individuals was estimated using the volume of intersection (VI) index [60]. This method measures similarity of two kernel utilisation distributions (UD) and thus compares not only area shared but also intensity of use [60]. The VI index ranges between 0 (no overlap) to 1 (identical UDs). The parameters used to calculate kernel UDs for all animals were: bandwidth h = 250 and grid size 200 based on visual assessment. Pairwise matrices of spatial overlap were used as a control for spatial proximity propensity when correlating association strength with genetic relatedness, and to compare space shared among animals forming social units. A correlation between social associations (HWI matrix) and genetic relatedness (pairwise relatedness matrix) was analysed using Mantel tests. The correlation was controlled for inter-individual spatial proximity using partial Mantel tests [55], [61], which determined the relationship between association and relatedness matrices while keeping the spatial overlap matrix constant. The significance of all correlations was assessed using 10.000 random permutations in SOCPROG. Genetic relatedness and spatial overlap between individuals within and across social units were compared with randomisation tests using 10.000 permutations to assess significance [62]. All spatial and statistical analyses were conducted using R version 2.13.1 software [63].

#### Temporal patterns of associations

Analysis of temporal stability of associations was based on GPS-telemetry data (fixes at 1-hour intervals) collected in 2010–2011 from 12 adult wild boar (6 males and 6 females) using lagged association rates (LAR) [31]. This technique provides a way to quantify the proportion and duration of short and long-term associations occurring in the population by calculating the probability that a pair of individuals recorded together at time zero will still be together at subsequent time periods, and averaging it over all associations. Each LAR was compared to the null association rate, expected if preferential associations do not occur. The uncertainty around the lagged association rates was estimated with a jackknifing procedure over 10-day periods [31]. A set of mathematical models approximating features of various social structures were fitted to the observed lagged association rates [31], [57]. The models utilise exponential decay and are composed of one, all, or any meaningful combination of three main components: constant companionships (permanent relationships lasting until death), casual acquaintances (associations lasting from a few days to a few years), and rapid disassociations (associations lasting a few hours at most). The best fitting and most parsimonious model was selected using quasi-Akaike Information Critrion corrected for a small sample size (qAIC_c_) [57]. The error around the model parameters approximating proportion and duration of different types of associations in the population was estimated using jackknifing. All analyses of the temporal association patterns were carried out in SOCPROG 2.4.

## Results

### Association Patterns and Social Network Structure

In the two years analysed, the majority of dyads did not associate (66% and 80%, respectively). The mean (± SE) values of the maximum HWI value for each individual (2008: 0.66±0.05; 2009: 0.50±0.05) indicated that some pairs of individuals formed strong associations and remained associated for 66% and 50% of the time in 2008 and 2009, respectively.

The observed mean HWI was different than the random mean (2008: observed mean = 0.095, random mean = 0.104, *p*<0.001, *n* = 465; 2009: observed mean = 0.068, random mean = 0.072, *p*<0.001, *n* = 435) and the observed coefficient of variation of the HWI was significantly higher than the random one (2008: observed CV = 2.54, random CV = 1.76, *p*<0.001; 2009: observed CV = 3.13, random CV = 2.44, *p*<0.001) indicating the presence of preferred associations and non-random character of the observed networks. The mean non-zero HWI was significantly greater than expected by chance (2008: observed mean = 0.277, random mean = 0.114, *p*<0.001; 2009: observed mean = 0.336, random mean = 0.110, *p*<0.001) and the proportion of non-zero associations in the population was significantly lower than expected by chance (2008: observed 34%, random 91%, *p*<0.001; 2009: observed 20%, random 66%, *p*<0.001) Hence, the wild boar in the study population had structured associations.

**Figure 1:**
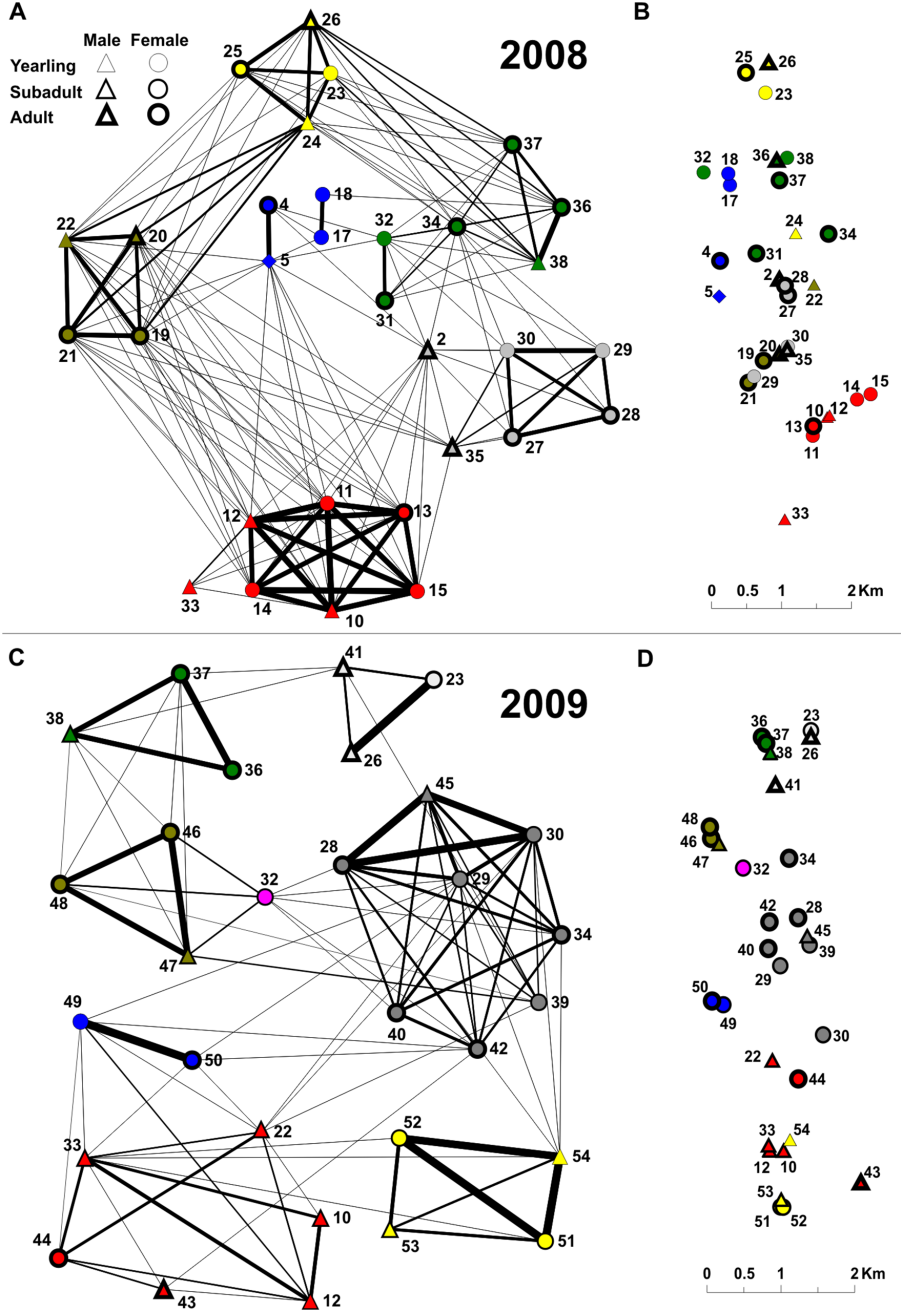
The social network of wild boar from Białowieża National Park, Poland. The network was constructed based on associations data in 2008 (A) and 2009 (C). Nodes and numbers symbolise individual animals, lines represent social ties. The thickness of the line corresponds to the strength of social bond. Colours represent social units determined by partitioning of the social network. Spatial distribution of the individuals within the study area in 2008 (B) and 2009 (D). Location of the individual’s symbol corresponds to its home range centre and colours indicate social units. Rhomb indicates individual with unidentified sex.

The resulting social networks were modular with 6 and 8 clusters (social units) in 2008 and 2009, respectively (Figure 1a, c). Modularity was maximised at 0.684 (2008) and 0.764 (2009) indicating strong division and marked structuring of the networks [64]. The average size of a social unit was 4.6±0.5 (mean ± SE) individuals. However, correcting social unit size for mark rate (55%) resulted in the expected social unit size of 8.4 individuals.

### Genetic and Spatial Effects on Network Structure

In total, 123 alleles were detected across 16 analysed loci. All loci were polymorphic with the number of alleles per locus ranging between 3 and 15 (Table 1). Missing data amounted 5.4% in the whole dataset and no individual was typed at less than 13 loci. Following sequential Bonferroni corrections, the overall population showed deviations from the Hardy-Weinberg equilibrium at two single loci (Table 1) and from linkage equilibrium in 10 (out of 120) pairs of loci. Analyses with the MICRO-CHECKER excluded problems associated with scoring errors, allelic dropout and null alleles (estimated frequency was <0.1 at all loci). No correlation between amount of homozygotes and amount of missing data was found neither across individuals (*r* = 0.025, *p* = 0.26) nor across loci (*r* = 0.184, *p* = 0.24). Thus, the observed deviations from equilibrium were most likely attributed to the inherent substructure of the population (i.e. presence of kin groups) and all loci were retained for statistical analyses. Overall, the coefficient of relatedness in the studied population averaged −0.002±0.001 (mean ± SE, *n* = 411 inds).

**Table 1:** Genetic variability of 16 microsatellite loci analysed in 411 wild boar from BPF.

Locus	Na	Allelic richness	H_e_	H_o_	HWE
					(*p*-value)
S090	8	6.52	0.687	0.654	0.054
SW72	6	4.48	0.655	0.649	0.345
S155	8	4.56	0.470	0.513	0.589
S026	4	3.80	0.510	0.536	0.583
S355	3	2.16	0.078	0.075	0.424
S215	3	2.95	0.223	0.220	0.797
SW951	6	2.72	0.038	0.021	0.012
SW857	5	4.13	0.642	0.614	**<0.001**
SW24	8	6.32	0.524	0.496	0.049
SW122	7	6.99	0.799	0.826	0.487
IGF1	11	9.21	0.833	0.847	0.206
SW461	10	9.92	0.867	0.888	0.008
SW1492	5	4.24	0.425	0.411	0.484
SW2021	15	11.22	0.828	0.829	0.051
SW2496	13	11.35	0.858	0.678	**<0.001**
SW2532	11	9.42	0.807	0.815	0.104
Mean (± SE)	7.7 (±0.89)	6.25 (±0.77)	0.578 (±0.069)	0.568 (±0.068)	

Na – observed number of alleles/locus, Allelic richness – mean number of alleles/locus over population, H_e_ – expected heterozygosity, H_o_ – observed heterozygosity, HWE (*p*-value) – probability of H_o_ given H_e_ (significant deviations from HWE following sequential Bonferroni correction are in bold).

In both 2008 and 2009, association strength and genetic relatedness were positively correlated (Table 2). The relationship was stronger in 2008, probably due to a higher proportion of yearlings (remaining within family groups) in the sampled animals compared to 2009 (52% and 3%, respectively). However, association strength and relatedness were also positively correlated when correlations were controlled for spatial overlap of utilised area, thus accounting for the family effect (Table 2). Association strength among females correlated positively with their genetic relatedness even when accounting for spatial overlap (Table 2). In contrast, association strength among males did not correlate with their relatedness, except in 2008 when not accounting for spatial overlap (Table 2). When spatial overlap was accounted for in this year, correlation among males disappeared suggesting a strong bias due to yearling males associated in family groups.

**Table 2:** Correlation coefficients between association strength and genetic relatedness in the wild boar population.

	2008			2009		
	*n*	*r*	*p*	*n*	*r*	*p*
All animals	465	0.502	<0.001	435	0.243	<0.001
Females	190	0.494	<0.001	136	0.210	0.007
Adult females	45	0.403	0.002	36	0.257	0.067
Males	45	0.325	0.020	78	0.131	0.134
Adult males	6	−0.136	0.587	3	-	-
Controlled for spatial overlap of utilised area						
All animals	465	0.209	<0.001	435	0.172	<0.001
Females	190	0.204	0.006	136	0.129	0.048
Adult females	45	0.308	0.015	36	0.357	0.017
Males	45	−0.032	0.569	78	0.172	0.086
Adult males	6	−0.439	0.829	3	-	-

Correlation coefficients (*r*) and statistical significance (*p*) were obtained using Mantel and partial Mantel (controlling for spatial overlap of utilised area) tests based on 10.000 permutations. Correlations for adult males in 2009 were not calculated due to low sample size. *n* – number of pairwise comparisons. See Table S1 for the relatedness and association matrix among all analysed individuals.

Both in 2008 and 2009, the degree of relatedness was higher among individuals within social units than between them (Table 3). Since similar patterns were observed in both years of the study, the data were pooled for sex-specific analysis. Adult females sharing membership of the social unit were more related among themselves than those belonging to different units (Table 3). In contrast, the degree of relatedness between adult females and adult males within and among social units did not differ (Table 3). The overlap of space utilisation distribution was significantly higher among individuals within social units than between them (Table 3). The same pattern held also true when only adult females and adult female – adult male dyads were considered (Table 3). Additionally, spatial overlap within social units (mean ± SE; 0.583±0.022) was markedly higher compared to the average overlap observed among all studied animals irrespective of the social unit membership (0.154±0.008). Spatial overlap was positively correlated with association strength (HWI) (Mantel test: *r* = 0.81, *n* = 900, *p*<0.001, 10 000 permutations). These results indicate that the spatial relationships of individuals were largely reflected in the social structure (Figure 1b, d). Genetic relatedness showed an evident sex-specific effect on the strength of social bond (Table 2) and social unit membership (Table 3).

**Table 3:** Mean (± SE) pairwise relatedness and spatial overlap between individuals in the wild boar social network.

	Social units				
	within	*n*	between	*n*	*p*
2008					
Relatedness	0.158±0.030	69	−0.013±0.009	399	<0.001
Spatial overlap	0.581±0.035	69	0.098±0.006	399	<0.001
2009					
Relatedness	0.078±0.028	59	0.001±0.010	380	0.004
Spatial overlap	0.584±0.030	59	0.065±0.006	380	<0.001
Both years					
Relatedness	0.122±0.022	128	−0.007±0.007	779	<0.001
Ad. F – ad. F	0.116±0.070	16	−0.020±0.020	87	0.008
Ad. F – ad. M	0.085±0.080	8	−0.025±0.025	67	0.068
Spatial overlap	0.583±0.022	128	0.082±0.004	779	<0.001
Ad. F – ad. F	0.593±0.058	16	0.089±0.012	87	<0.001
Ad. F – ad. M	0.502±0.091	8	0.108±0.016	67	<0.001

Average relatedness and spatial overlap are given for individuals sharing membership of the social unit (within) and those associated with different units (between). Social units result from network partitioning based solely on associations frequency (see Figure 1). Statistical significance of the differences was obtained with randomisation tests based on 10.000 permutations. *n* – number of dyads. See Table S1 for the relatedness and spatial overlap matrix among all analysed individuals.

### Temporal Patterns of Associations

**Figure 2:**
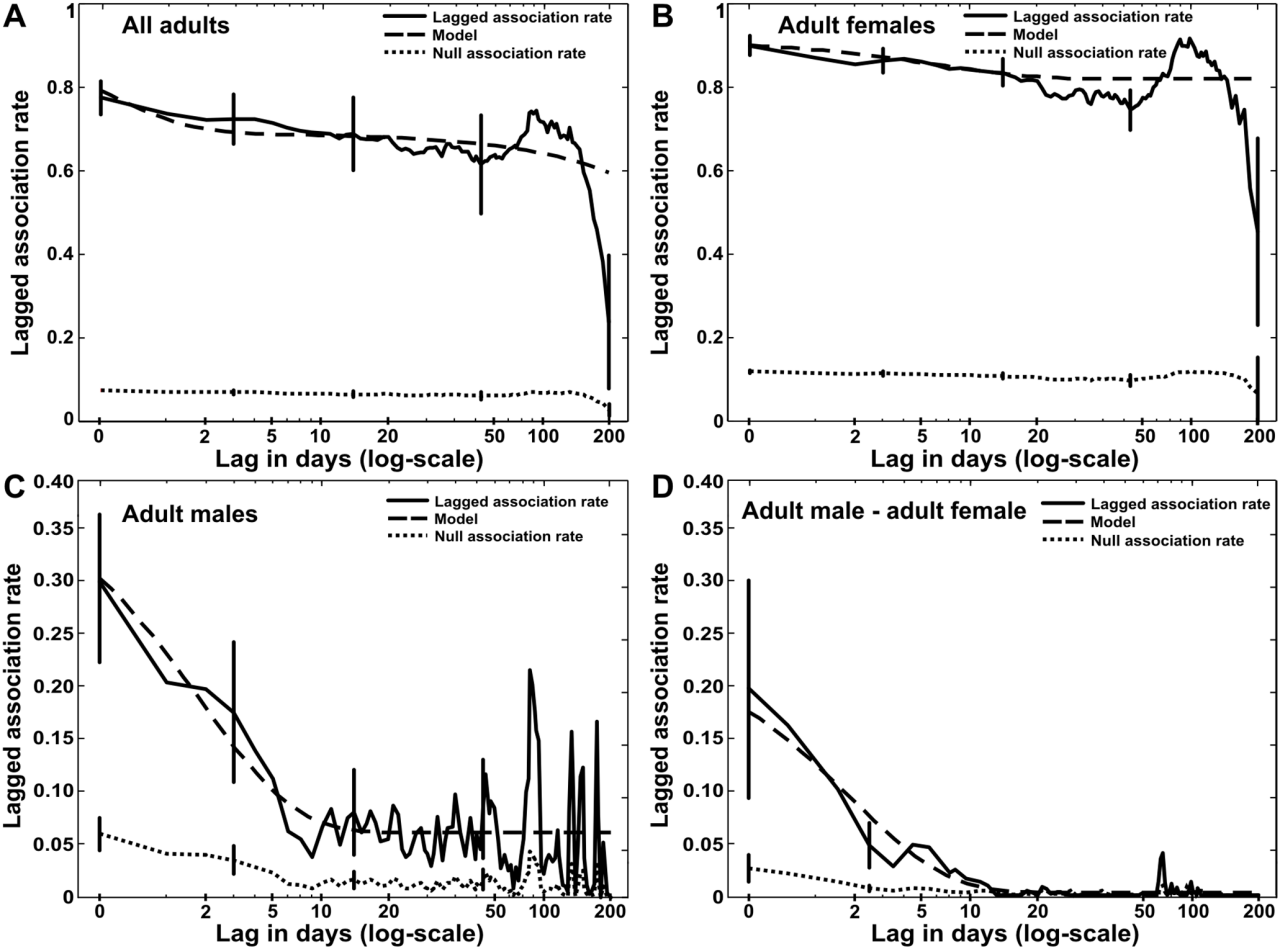
Temporal patterns of wild boar associations. Stability of associations were estimated using lagged association rates (LARs). The LARs were compared to null association rates (LAR if individuals associated randomly) and the best fitting model is shown for each LAR (see Table 4 for description). Standard error bars were obtained by jackknifing.

Adult wild boar formed non-random and temporarily stable associations (Figure 2a). The levels of LAR were higher than expected by chance and did not fall to null association level (i.e. LAR if individuals associated randomly). Interaction patterns were dominated by long-term relationships which lasted a few years and represented 69% of the associations in the population (Table 4). Short-term, casual acquaintances lasting on average one day characterised roughly one-third of the associations (Table 4). However, there were strong sexual differences in temporal pattern of associations. Associations of adult females were particularly long-lasting (Figure 2b). The majority (81%) of female-female associations were potentially lifelong while the rest lasted for about a week (10% of associations) or disintegrated within a day (Table 4). Conversely, male–male and male–female relationships were more dynamic and reached the level of random association after a relatively short time (Figure 2c and 2d). Most of associations among adult males (60%) broke down within a day, 34% lasted several days, and only 6% had permanent character (Table 4). Male–female interactions were particularly short-lived: 75.8% of associations disintegrated within a day, 24% lasted a few days and 0.2% were long-lasting relationships (Table 4).

**Table 4:** Proportions and temporal characteristics of the social components in the wild boar population.

Model component	Value	(SE range)
All adults		
Permanent acquaintances	69%	(60–78)
Duration of permanent acquaintances	3.7 years	(1.6–9.1)
Casual acquaintances	31%	(22–40)
Duration of casual acquaintances	0.9 days	(0.5–4.8)
Female – female		
Constant companionships	81%	(72–90)
Casual acquaintances	10%	(4–16)
Duration of casual acquaintances	7.1 days	(4.4–18.4)
Male – male		
Constant companionships	6%	(5–7)
Casual acquaintances	34%	(13–55)
Duration of casual acquaintances	2.8 days	(2.0–4.6)
Male – female		
Constant companionships	0.2%	(0–0.8)
Casual acquaintances	24%	(3–45)
Duration of casual acquaintances	2.9 days	(1.1–4.8)

The models fitted to the lagged association rates (LARs; see Figure 2) consist of a proportion of constant companions, rapid disassociations, and casual acquaintances of two types: permanent acquaintances lasting for particular period of time and casual acquaintances that last for shorter periods. This values correspond to percentage of each social component in the population. The standard error (SE range around the mean) of each parameter was estimated by jackknifing procedure. For a more detailed description and formulation of the models see Whitehead 1999 [31] and 2008 [57].

## Discussion

Wild boar in the study population formed non-random, preferential associations. The majority of dyads did not associate, whereas some pairs of individuals formed strong associations, spending over half of their time together. Although studies allowing comparison with other wild boar populations are lacking, such association patterns are expected for group-living animals [28], [34].

The correlation between association strength and genetic relatedness indicates that wild boars in BPF spend more time with individuals to which they are more related. This could have merely been an effect of the spatial distribution of individuals, i.e. animals closer to each other having a greater chance of interacting with kin neighbours due to cross-generational site fidelity. However, the positive relationship between the strength of social bond and relatedness held true when accounting for potential bias caused by spatial proximity. This indicated the presence of targeted interactions among kin. The behavioural mechanisms and benefits of these associations in wild boar are not well understood. If inclusive fitness benefits are the main drivers of targeted kin interactions in a matrilineal systems, we would expect interactions among related females to be favoured in wild boar. Indeed, the data showed that females associated preferentially with related females, even when accounting for spatial proximity. This result provides evidence that kin-targeted interactions among females underlie the observed kin structures, which are thus not entirely the result of a simplistic, passive process of local accumulation of relatedness. Social bonds between related females have been demonstrated to have a positive effect on female fitness, including increased offspring survival, in other group-living species [65]–[68]. In contrast to females, wild boar males, particularly adults, tended to form associations with unrelated males which seems to conform with polygynous mating system and male-biased dispersal in this species [21], [22]. However, given the low number of adult males in this study and the potential bias in relatedness estimates [24], [25], this result should be treated with caution.

Our results underline the central role of females in wild boar social system and conform to previous studies describing matrilineality in this species [20], [21]. Multigenerational and female-dominated social units can be advantageous for females to optimise foraging and rearing of young when multiple litters are present simultaneously in a group. Wild boars exhibit a high synchrony of reproduction within one social group [69] and produce large litters [70], hence cooperative breeding may play important role in shaping the observed social structure [20]. Additionally, winter severity and food abundance are the major factors affecting the reproductive performance of wild boar females in the temperate zone [71], [72]. Therefore, achieving good physical condition and gaining sufficient fat reserves before winter is crucial for female wild boar fitness. In our study area, the acorn crop occurring in autumn is the most efficient way to achieve the above [71]. Individual oaks show high variation in acorn production ([73]; T. Podgórski, unpublished data), creating a heterogeneous distribution of food resources in this crucial period. Therefore, acquiring information on high quality food patches would be advantageous to young, inexperienced females and this would reinforce interactions among related females and encourage philopatry. Foraging efficiency can be considerably improved by information obtained through social learning [74], [75] and use of spatial memory [76]. The prediction that such mechanisms shape wild boar sociality needs to be further tested.

Repeated and non-random interactions favour cooperative behaviours and facilitate behavioural reciprocity [77] leading to strong bonds between some animals. Site fidelity occurring over generations result in local clustering of kin or matrilines [78]–[80] and increase the chance of frequent interactions with relatives. In such a scenario, likely to be present in female wild boar which are philopatric [22], strong social bonds between relatives can be favoured due to increased indirect fitness benefits [66]–[68], [79]. Our results, showing temporarily stable and kin-targeted females associations, hint at the important role of kin selection in shaping social relationships among female wild boar. Interestingly, interactions among kin were not a major factor shaping wild boar sociality in the heavily harvested population where large proportion of females was removed annually [23]. This contrasting results raise questions about indirect social effects of removal and their consequences for population dynamics which require further comparative studies. In contrast to females in our study, associations of adult males (with other males and females) were dynamic and short-lived, which is consistent with the solitary lifestyle of adult male boars described previously [17], [19], [81]. The majority (65–75%) of male’s associations disintegrated within a day and the rest lasted a few days at most. Short-time casual acquaintances, in which adult males engage, may be due to interactions with mating competitors (associations with other males), assessment of females reproductive status (with females), or enhancement of foraging efficiency by the utilising social cues provided by groups (with females and/or groups).

Genetic structure can emerge as a by-product of philopatry through a passive process of localised relatedness accumulation [79], [80]. However, spatial segregation in this study did not entirely explain the observed kin-based structure. Indeed, our fine-scale analysis of association preferences showed that spatial segregation did not fully account for the observed grouping patterns. The majority of marked individuals (82%) showed some overlap and thus, potentially, they had the chance to interact. However, only 26% of animals associated at least once. Furthermore, some pairs of individuals sharing as much as 40–50% of utilised area did not form associations, and some pairs associated infrequently (half-weight association index ≤0.22) despite extensive spatial overlap of their utilised area (66–79%). Finally, genetic data showed that preferential, kin targeted, associations persisted in the population regardless of spatial proximity. Our results show that kin-directed social preferences in wild boar extend beyond simple spatial proximity and direct mother-offspring ties within groups and thus imply the potential role of kin recognition as a mechanism driving choice of a social partners.

This study demonstrated, for the first time in wild boar, how social preferences of individuals translate into an emergent socio-genetic population structure. Wild boar population was organised into spatially and genetically structured social units. Genuine, kin-targeted social interactions of females were temporarily stable and extended beyond spatial proximity between individuals, underlying observed social organisation. Given the natural environment of the study population and its minimally disturbed character, we believe that the observed patterns of social relationships represent a picture of reference of the social structure of wild boar inhabiting the forests of the European temperate zone.

## Supporting Information

Table S1 **Matrix of relatedness, association index and spatial overlap among individuals in two years (2008 and 2009) of the study.**
(PDF)
