# Supplementary material for: Long-Lasting, Kin-Directed Female Interactions in a Spatially Structured Wild Boar Social Network
Source: PLoS One. 2014 Jun 11;9(6):e99875. doi: 10.1371/journal.pone.0099875 (PMC4053407; doi:10.1371/journal.pone.0099875)
Supplement: Table S1 — Matrix of relatedness, association index and spatial overlap among individuals in two years (2008 and 2009) of the study. (PDF) [file pone.0099875.s001.pdf]

Table S1. Matrix of relatedness (Queller & Goodnight 1989), association index (HWI) and spatial overlap (VI index) among individuals in two years (2008 and 2009) of the study. Animal IDs (ID_1 and ID_2) correspond to the numbers in Figure 1.

Year	ID_1	ID_2	Relatedness	Association index (HWI)	Spatial overlap (VI index)
2008	2	4	0.411	0.04	0.249
2008	2	5	0.117	0.07	0.237
2008	2	10	-0.208	0.03	0.184
2008	2	11	-0.171	0.01	0.169
2008	2	12	-0.252	0.01	0.171
2008	2	13	0.078	0.01	0.183
2008	2	14	-0.045	0.02	0.225
2008	2	15	-0.041	0.02	0.210
2008	2	17	-0.043	0.00	0.240
2008	2	18	0.120	0.00	0.172
2008	2	19	0.056	0.00	0.140
2008	2	20	0.199	0.00	0.149
2008	2	21	0.062	0.00	0.145
2008	2	22	0.092	0.00	0.139
2008	2	23	-0.105	0.00	0.040
2008	2	24	-0.147	0.00	0.080
2008	2	25	0.091	0.00	0.081
2008	2	26	0.380	0.00	0.054
2008	2	27	-0.046	0.05	0.373
2008	2	28	-0.044	0.06	0.326
2008	2	29	0.125	0.05	0.338
2008	2	30	0.103	0.04	0.303
2008	2	31	-0.030	0.00	0.372
2008	2	32	0.200	0.02	0.454
2008	2	33	0.198	0.00	0.013
2008	2	34	0.196	0.04	0.410
2008	2	35	-0.060	0.07	0.367
2008	2	36	-0.001	0.00	0.242
2008	2	37	0.287	0.00	0.242
2008	2	38	-0.032	0.00	0.230
2008	4	5	0.053	0.78	0.947
2008	4	10	-0.080	0.00	0.002
2008	4	11	-0.094	0.00	0.001
2008	4	12	-0.225	0.00	0.002
2008	4	13	-0.283	0.00	0.003
2008	4	14	-0.068	0.00	0.002
2008	4	15	-0.181	0.00	0.002
2008	4	17	-0.278	0.03	0.145
2008	4	18	-0.189	0.00	0.073
2008	4	19	-0.042	0.00	0.020

2008	4	20	0.239	0.00	0.021
2008	4	21	0.023	0.00	0.022
2008	4	22	0.018	0.00	0.025
2008	4	23	-0.255	0.00	0.002
2008	4	24	-0.344	0.00	0.010
2008	4	25	-0.350	0.00	0.006
2008	4	26	-0.190	0.00	0.002
2008	4	27	-0.152	0.00	0.120
2008	4	28	-0.024	0.00	0.093
2008	4	29	-0.116	0.00	0.081
2008	4	30	0.084	0.00	0.067
2008	4	31	-0.077	0.00	0.257
2008	4	32	0.048	0.02	0.234
2008	4	33	0.143	0.00	0.001
2008	4	34	0.087	0.00	0.101
2008	4	35	-0.085	0.00	0.094
2008	4	36	0.160	0.00	0.072
2008	4	37	0.159	0.00	0.045
2008	4	38	0.166	0.00	0.074
2008	5	10	0.092	0.01	0.017
2008	5	11	-0.090	0.01	0.017
2008	5	12	0.203	0.01	0.017
2008	5	13	-0.042	0.01	0.019
2008	5	14	-0.022	0.00	0.017
2008	5	15	0.126	0.02	0.018
2008	5	17	-0.124	0.02	0.128
2008	5	18	0.116	0.00	0.063
2008	5	19	0.245	0.01	0.035
2008	5	20	0.229	0.00	0.036
2008	5	21	0.255	0.01	0.037
2008	5	22	0.116	0.01	0.039
2008	5	23	-0.107	0.00	0.001
2008	5	24	0.106	0.00	0.024
2008	5	25	-0.201	0.00	0.005
2008	5	26	-0.242	0.00	0.002
2008	5	27	0.021	0.00	0.118
2008	5	28	0.102	0.00	0.095
2008	5	29	0.086	0.00	0.083
2008	5	30	0.155	0.00	0.071
2008	5	31	0.051	0.00	0.244
2008	5	32	0.254	0.02	0.221
2008	5	33	-0.026	0.00	0.016
2008	5	34	0.051	0.00	0.095
2008	5	35	0.434	0.00	0.108
2008	5	36	0.243	0.00	0.069
2008	5	37	0.094	0.00	0.041

2008	5	38	0.179	0.00	0.071
2008	10	11	0.504	0.97	0.893
2008	10	12	0.415	1.02	0.906
2008	10	13	0.358	0.81	0.855
2008	10	14	0.462	0.84	0.724
2008	10	15	0.465	0.93	0.819
2008	10	17	0.021	0.00	0.000
2008	10	18	0.166	0.00	0.000
2008	10	19	0.227	0.05	0.288
2008	10	20	-0.091	0.03	0.216
2008	10	21	0.066	0.06	0.291
2008	10	22	0.213	0.03	0.240
2008	10	23	-0.122	0.00	0.000
2008	10	24	-0.089	0.00	0.066
2008	10	25	-0.259	0.00	0.000
2008	10	26	-0.039	0.00	0.000
2008	10	27	0.105	0.00	0.039
2008	10	28	0.273	0.00	0.074
2008	10	29	0.073	0.00	0.070
2008	10	30	0.108	0.00	0.094
2008	10	31	0.032	0.00	0.005
2008	10	32	-0.082	0.00	0.007
2008	10	33	-0.052	0.06	0.365
2008	10	34	0.127	0.00	0.035
2008	10	35	0.078	0.00	0.161
2008	10	36	-0.058	0.00	0.003
2008	10	37	-0.086	0.00	0.005
2008	10	38	-0.055	0.00	0.002
2008	11	12	0.371	0.99	0.865
2008	11	13	0.480	0.84	0.865
2008	11	14	0.626	0.80	0.684
2008	11	15	0.267	0.88	0.780
2008	11	17	0.087	0.00	0.000
2008	11	18	0.228	0.00	0.000
2008	11	19	-0.014	0.04	0.255
2008	11	20	-0.405	0.01	0.190
2008	11	21	-0.138	0.05	0.262
2008	11	22	0.018	0.02	0.200
2008	11	23	-0.167	0.00	0.000
2008	11	24	-0.032	0.00	0.063
2008	11	25	-0.112	0.00	0.000
2008	11	26	0.222	0.00	0.000
2008	11	27	0.126	0.00	0.032
2008	11	28	0.111	0.00	0.071
2008	11	29	-0.217	0.00	0.061
2008	11	30	0.118	0.00	0.085

2008	11	31	-0.193	0.00	0.004
2008	11	32	-0.219	0.00	0.006
2008	11	33	-0.079	0.06	0.368
2008	11	34	-0.004	0.00	0.031
2008	11	35	-0.121	0.00	0.137
2008	11	36	-0.083	0.00	0.003
2008	11	37	-0.223	0.00	0.005
2008	11	38	0.081	0.00	0.002
2008	12	13	0.346	0.86	0.878
2008	12	14	0.186	0.80	0.691
2008	12	15	0.228	0.90	0.800
2008	12	17	0.029	0.00	0.000
2008	12	18	0.226	0.00	0.000
2008	12	19	0.428	0.05	0.288
2008	12	20	0.157	0.03	0.216
2008	12	21	0.131	0.07	0.294
2008	12	22	0.254	0.02	0.241
2008	12	23	-0.153	0.00	0.000
2008	12	24	-0.063	0.00	0.067
2008	12	25	-0.125	0.00	0.000
2008	12	26	0.216	0.00	0.000
2008	12	27	-0.191	0.00	0.037
2008	12	28	0.045	0.00	0.073
2008	12	29	-0.087	0.00	0.066
2008	12	30	-0.258	0.00	0.091
2008	12	31	-0.070	0.00	0.004
2008	12	32	0.132	0.00	0.006
2008	12	33	-0.090	0.08	0.404
2008	12	34	-0.139	0.00	0.032
2008	12	35	0.249	0.00	0.159
2008	12	36	-0.138	0.00	0.003
2008	12	37	-0.064	0.00	0.004
2008	12	38	-0.021	0.00	0.002
2008	13	14	0.505	0.71	0.694
2008	13	15	0.396	0.83	0.805
2008	13	17	-0.159	0.00	0.000
2008	13	18	0.334	0.00	0.000
2008	13	19	0.413	0.03	0.304
2008	13	20	0.077	0.03	0.230
2008	13	21	0.226	0.05	0.313
2008	13	22	0.194	0.03	0.253
2008	13	23	-0.010	0.00	0.000
2008	13	24	0.140	0.00	0.084
2008	13	25	-0.056	0.00	0.000
2008	13	26	0.203	0.00	0.000
2008	13	27	0.176	0.00	0.039

2008	13	28	0.324	0.00	0.074
2008	13	29	0.122	0.00	0.068
2008	13	30	0.082	0.00	0.094
2008	13	31	0.016	0.00	0.004
2008	13	32	0.173	0.00	0.006
2008	13	33	-0.162	0.03	0.330
2008	13	34	-0.036	0.00	0.031
2008	13	35	0.118	0.00	0.176
2008	13	36	-0.138	0.00	0.003
2008	13	37	-0.167	0.00	0.004
2008	13	38	0.002	0.00	0.002
2008	14	15	0.275	0.98	0.857
2008	14	17	-0.218	0.00	0.000
2008	14	18	0.273	0.00	0.000
2008	14	19	0.319	0.03	0.259
2008	14	20	-0.199	0.02	0.235
2008	14	21	0.009	0.03	0.274
2008	14	22	0.144	0.01	0.250
2008	14	23	0.013	0.00	0.000
2008	14	24	-0.134	0.00	0.061
2008	14	25	-0.080	0.00	0.000
2008	14	26	0.108	0.00	0.000
2008	14	27	0.283	0.00	0.054
2008	14	28	0.207	0.00	0.052
2008	14	29	0.019	0.00	0.076
2008	14	30	0.231	0.00	0.080
2008	14	31	-0.083	0.00	0.006
2008	14	32	-0.033	0.00	0.009
2008	14	33	-0.132	0.00	0.146
2008	14	34	0.042	0.00	0.045
2008	14	35	-0.143	0.00	0.203
2008	14	36	-0.013	0.00	0.004
2008	14	37	-0.132	0.00	0.006
2008	14	38	0.162	0.00	0.003
2008	15	17	-0.223	0.00	0.000
2008	15	18	0.135	0.00	0.000
2008	15	19	0.343	0.03	0.294
2008	15	20	-0.057	0.02	0.244
2008	15	21	0.040	0.04	0.301
2008	15	22	0.359	0.03	0.264
2008	15	23	-0.231	0.00	0.000
2008	15	24	-0.006	0.00	0.073
2008	15	25	-0.214	0.00	0.000
2008	15	26	-0.132	0.00	0.000
2008	15	27	0.128	0.00	0.048
2008	15	28	0.119	0.00	0.080

2008	15	29	0.100	0.00	0.078
2008	15	30	-0.076	0.00	0.106
2008	15	31	0.130	0.00	0.006
2008	15	32	-0.054	0.00	0.008
2008	15	33	-0.214	0.00	0.245
2008	15	34	-0.077	0.00	0.039
2008	15	35	0.111	0.04	0.189
2008	15	36	0.014	0.00	0.004
2008	15	37	-0.188	0.00	0.006
2008	15	38	-0.158	0.00	0.003
2008	17	18	0.135	0.70	0.835
2008	17	19	-0.274	0.00	0.001
2008	17	20	-0.144	0.00	0.001
2008	17	21	-0.454	0.00	0.002
2008	17	22	-0.283	0.00	0.001
2008	17	23	-0.222	0.00	0.022
2008	17	24	-0.183	0.00	0.017
2008	17	25	-0.252	0.00	0.050
2008	17	26	-0.123	0.00	0.036
2008	17	27	-0.004	0.00	0.043
2008	17	28	-0.300	0.00	0.045
2008	17	29	-0.269	0.00	0.028
2008	17	30	-0.029	0.00	0.015
2008	17	31	0.016	0.00	0.109
2008	17	32	-0.272	0.00	0.206
2008	17	33	0.049	0.00	0.000
2008	17	34	0.510	0.00	0.094
2008	17	35	-0.234	0.00	0.032
2008	17	36	0.086	0.00	0.196
2008	17	37	-0.120	0.00	0.178
2008	17	38	0.013	0.00	0.185
2008	18	19	0.020	0.00	0.001
2008	18	20	-0.007	0.00	0.001
2008	18	21	-0.154	0.00	0.002
2008	18	22	-0.038	0.00	0.001
2008	18	23	-0.103	0.00	0.022
2008	18	24	-0.262	0.00	0.016
2008	18	25	-0.288	0.00	0.049
2008	18	26	-0.026	0.00	0.037
2008	18	27	0.167	0.00	0.031
2008	18	28	0.053	0.00	0.036
2008	18	29	-0.032	0.00	0.021
2008	18	30	0.175	0.00	0.011
2008	18	31	0.056	0.00	0.060
2008	18	32	0.105	0.00	0.153
2008	18	33	-0.123	0.00	0.000

2008	18	34	0.443	0.00	0.063
2008	18	35	-0.020	0.00	0.025
2008	18	36	-0.051	0.02	0.189
2008	18	37	0.046	0.00	0.158
2008	18	38	-0.174	0.00	0.178
2008	19	20	0.461	0.56	0.830
2008	19	21	0.280	0.82	0.904
2008	19	22	0.606	0.60	0.878
2008	19	23	-0.123	0.01	0.025
2008	19	24	0.131	0.20	0.242
2008	19	25	-0.240	0.01	0.025
2008	19	26	-0.036	0.01	0.025
2008	19	27	-0.011	0.00	0.078
2008	19	28	0.045	0.00	0.077
2008	19	29	-0.073	0.00	0.119
2008	19	30	-0.080	0.00	0.116
2008	19	31	-0.002	0.00	0.026
2008	19	32	0.285	0.00	0.023
2008	19	33	-0.141	0.00	0.139
2008	19	34	-0.073	0.00	0.044
2008	19	35	0.292	0.02	0.446
2008	19	36	0.189	0.00	0.033
2008	19	37	0.009	0.00	0.034
2008	19	38	-0.085	0.00	0.032
2008	20	21	0.388	0.62	0.847
2008	20	22	0.179	0.60	0.894
2008	20	23	-0.057	0.00	0.017
2008	20	24	-0.220	0.23	0.237
2008	20	25	-0.367	0.00	0.017
2008	20	26	-0.210	0.00	0.017
2008	20	27	-0.139	0.00	0.083
2008	20	28	0.036	0.00	0.069
2008	20	29	-0.051	0.00	0.101
2008	20	30	-0.150	0.00	0.106
2008	20	31	0.114	0.00	0.030
2008	20	32	0.444	0.00	0.026
2008	20	33	0.048	0.00	0.097
2008	20	34	-0.064	0.00	0.038
2008	20	35	0.215	0.02	0.491
2008	20	36	-0.007	0.00	0.025
2008	20	37	0.029	0.00	0.028
2008	20	38	-0.111	0.00	0.025
2008	21	22	0.044	0.64	0.874
2008	21	23	0.047	0.00	0.027
2008	21	24	0.013	0.21	0.248
2008	21	25	-0.180	0.01	0.027

2008	21	26	-0.005	0.01	0.027
2008	21	27	-0.020	0.00	0.087
2008	21	28	0.320	0.00	0.089
2008	21	29	0.244	0.00	0.124
2008	21	30	-0.008	0.00	0.126
2008	21	31	-0.039	0.00	0.037
2008	21	32	0.404	0.00	0.030
2008	21	33	-0.073	0.00	0.146
2008	21	34	-0.293	0.00	0.047
2008	21	35	0.360	0.02	0.460
2008	21	36	-0.019	0.00	0.035
2008	21	37	0.066	0.00	0.038
2008	21	38	-0.206	0.00	0.035
2008	22	23	-0.251	0.01	0.023
2008	22	24	-0.101	0.20	0.243
2008	22	25	-0.275	0.01	0.023
2008	22	26	0.078	0.01	0.023
2008	22	27	0.017	0.00	0.077
2008	22	28	-0.109	0.00	0.062
2008	22	29	-0.008	0.00	0.094
2008	22	30	0.065	0.00	0.097
2008	22	31	0.012	0.00	0.031
2008	22	32	0.097	0.00	0.026
2008	22	33	-0.129	0.00	0.098
2008	22	34	-0.060	0.00	0.036
2008	22	35	0.215	0.02	0.471
2008	22	36	0.059	0.00	0.032
2008	22	37	-0.155	0.00	0.033
2008	22	38	-0.114	0.00	0.032
2008	23	24	0.467	0.49	0.718
2008	23	25	0.281	0.54	0.747
2008	23	26	-0.063	0.54	0.748
2008	23	27	-0.100	0.00	0.007
2008	23	28	0.003	0.00	0.053
2008	23	29	-0.092	0.00	0.013
2008	23	30	-0.113	0.00	0.001
2008	23	31	0.011	0.00	0.092
2008	23	32	0.199	0.00	0.068
2008	23	33	0.025	0.00	0.000
2008	23	34	-0.170	0.04	0.168
2008	23	35	-0.257	0.00	0.001
2008	23	36	-0.250	0.07	0.443
2008	23	37	-0.252	0.07	0.447
2008	23	38	-0.108	0.08	0.464
2008	24	25	0.226	0.58	0.735
2008	24	26	-0.144	0.50	0.696

2008	24	27	0.026	0.00	0.023
2008	24	28	0.032	0.00	0.063
2008	24	29	-0.059	0.00	0.031
2008	24	30	0.024	0.00	0.021
2008	24	31	-0.155	0.00	0.097
2008	24	32	-0.015	0.00	0.068
2008	24	33	0.034	0.00	0.051
2008	24	34	-0.223	0.02	0.167
2008	24	35	0.087	0.00	0.135
2008	24	36	-0.157	0.04	0.401
2008	24	37	-0.335	0.03	0.447
2008	24	38	-0.072	0.04	0.404
2008	25	26	0.025	0.60	0.714
2008	25	27	-0.118	0.00	0.017
2008	25	28	-0.180	0.00	0.067
2008	25	29	-0.066	0.00	0.021
2008	25	30	-0.138	0.00	0.002
2008	25	31	-0.268	0.00	0.135
2008	25	32	-0.201	0.00	0.106
2008	25	33	-0.137	0.00	0.000
2008	25	34	-0.370	0.03	0.233
2008	25	35	-0.171	0.00	0.004
2008	25	36	-0.373	0.07	0.483
2008	25	37	-0.434	0.06	0.531
2008	25	38	-0.464	0.07	0.474
2008	26	27	-0.183	0.00	0.008
2008	26	28	-0.096	0.00	0.047
2008	26	29	-0.114	0.00	0.013
2008	26	30	-0.224	0.00	0.001
2008	26	31	-0.320	0.00	0.089
2008	26	32	0.054	0.00	0.070
2008	26	33	0.022	0.00	0.000
2008	26	34	-0.190	0.03	0.180
2008	26	35	-0.204	0.00	0.001
2008	26	36	-0.283	0.09	0.474
2008	26	37	0.071	0.08	0.459
2008	26	38	-0.054	0.11	0.492
2008	27	28	0.456	0.68	0.849
2008	27	29	0.279	0.52	0.775
2008	27	30	0.652	0.55	0.796
2008	27	31	0.154	0.00	0.348
2008	27	32	0.174	0.00	0.339
2008	27	33	0.097	0.00	0.003
2008	27	34	0.155	0.03	0.418
2008	27	35	0.014	0.07	0.409
2008	27	36	-0.007	0.00	0.089

2008	27	37	-0.048	0.00	0.098
2008	27	38	-0.071	0.00	0.083
2008	28	29	0.605	0.53	0.737
2008	28	30	0.117	0.50	0.775
2008	28	31	0.102	0.00	0.344
2008	28	32	0.444	0.00	0.334
2008	28	33	-0.054	0.00	0.048
2008	28	34	-0.049	0.03	0.425
2008	28	35	0.084	0.04	0.344
2008	28	36	-0.063	0.00	0.138
2008	28	37	0.027	0.00	0.149
2008	28	38	-0.209	0.00	0.134
2008	29	30	0.218	0.78	0.893
2008	29	31	0.082	0.00	0.243
2008	29	32	0.323	0.03	0.233
2008	29	33	-0.204	0.00	0.021
2008	29	34	0.056	0.06	0.344
2008	29	35	0.129	0.08	0.448
2008	29	36	-0.132	0.00	0.080
2008	29	37	-0.057	0.00	0.090
2008	29	38	-0.249	0.00	0.076
2008	30	31	-0.097	0.00	0.217
2008	30	32	-0.098	0.02	0.209
2008	30	33	0.190	0.00	0.052
2008	30	34	0.244	0.07	0.321
2008	30	35	0.054	0.08	0.430
2008	30	36	-0.132	0.00	0.051
2008	30	37	-0.057	0.00	0.063
2008	30	38	-0.249	0.00	0.049
2008	31	32	0.330	0.57	0.800
2008	31	33	-0.206	0.00	0.000
2008	31	34	-0.063	0.08	0.372
2008	31	35	-0.087	0.00	0.187
2008	31	36	-0.214	0.02	0.227
2008	31	37	-0.273	0.02	0.291
2008	31	38	-0.253	0.02	0.209
2008	32	33	-0.020	0.00	0.000
2008	32	34	-0.119	0.10	0.406
2008	32	35	0.193	0.00	0.162
2008	32	36	-0.013	0.02	0.232
2008	32	37	0.194	0.04	0.280
2008	32	38	-0.146	0.02	0.215
2008	33	34	0.060	0.00	0.000
2008	33	35	-0.105	0.00	0.052
2008	33	36	-0.214	0.00	0.000
2008	33	37	0.065	0.00	0.000

2008	33	38	-0.151	0.00	0.000
2008	34	35	-0.219	0.00	0.234
2008	34	36	0.328	0.12	0.304
2008	34	37	0.245	0.10	0.335
2008	34	38	0.144	0.10	0.290
2008	35	36	-0.005	0.00	0.046
2008	35	37	-0.036	0.00	0.053
2008	35	38	-0.189	0.00	0.045
2008	36	37	0.198	0.34	0.681
2008	36	38	0.294	0.85	0.897
2008	37	38	-0.065	0.41	0.656
2009	10	12	0.415	0.36	0.728
2009	10	22	0.213	0.00	0.289
2009	10	23	-0.122	0.00	0.000
2009	10	26	-0.039	0.00	0.000
2009	10	28	0.273	0.00	0.001
2009	10	29	0.073	0.00	0.030
2009	10	30	0.108	0.00	0.004
2009	10	32	-0.082	0.00	0.000
2009	10	33	-0.052	0.22	0.743
2009	10	34	0.127	0.00	0.000
2009	10	36	-0.058	0.00	0.000
2009	10	37	-0.086	0.00	0.000
2009	10	38	-0.055	0.00	0.000
2009	10	39	-0.125	0.00	0.007
2009	10	40	-0.187	0.00	0.019
2009	10	41	0.278	0.00	0.000
2009	10	42	0.072	0.00	0.000
2009	10	43	-0.140	0.00	0.152
2009	10	44	-0.006	0.00	0.335
2009	10	45	0.396	0.00	0.001
2009	10	46	-0.040	0.00	0.000
2009	10	47	-0.077	0.00	0.000
2009	10	48	0.033	0.00	0.000
2009	10	49	-0.076	0.00	0.061
2009	10	50	-0.089	0.00	0.040
2009	10	51	0.148	0.00	0.534
2009	10	52	-0.449	0.00	0.509
2009	10	53	-0.222	0.00	0.425
2009	10	54	-0.087	0.00	0.512
2009	12	22	0.254	0.15	0.343
2009	12	23	-0.153	0.00	0.000
2009	12	26	0.216	0.00	0.000
2009	12	28	0.045	0.00	0.001
2009	12	29	-0.087	0.00	0.030
2009	12	30	-0.258	0.00	0.003

2009	12	32	0.132	0.00	0.000
2009	12	33	-0.090	0.39	0.813
2009	12	34	-0.139	0.00	0.000
2009	12	36	-0.138	0.00	0.000
2009	12	37	-0.064	0.00	0.000
2009	12	38	-0.021	0.00	0.000
2009	12	39	-0.163	0.00	0.005
2009	12	40	0.150	0.00	0.021
2009	12	41	-0.015	0.00	0.000
2009	12	42	-0.296	0.00	0.000
2009	12	43	-0.179	0.00	0.125
2009	12	44	0.310	0.03	0.270
2009	12	45	0.027	0.00	0.001
2009	12	46	0.033	0.00	0.000
2009	12	47	-0.012	0.00	0.000
2009	12	48	0.095	0.00	0.000
2009	12	49	0.038	0.06	0.106
2009	12	50	-0.176	0.00	0.075
2009	12	51	0.359	0.00	0.425
2009	12	52	-0.069	0.00	0.430
2009	12	53	-0.140	0.00	0.359
2009	12	54	0.109	0.00	0.423
2009	22	23	-0.251	0.00	0.000
2009	22	26	0.078	0.00	0.000
2009	22	28	-0.109	0.00	0.048
2009	22	29	-0.008	0.00	0.100
2009	22	30	0.065	0.02	0.079
2009	22	32	0.097	0.00	0.003
2009	22	33	-0.129	0.17	0.387
2009	22	34	-0.060	0.00	0.024
2009	22	36	0.059	0.00	0.000
2009	22	37	-0.155	0.00	0.000
2009	22	38	-0.114	0.00	0.000
2009	22	39	0.057	0.04	0.111
2009	22	40	0.108	0.00	0.069
2009	22	41	0.065	0.00	0.009
2009	22	42	0.107	0.00	0.040
2009	22	43	-0.354	0.00	0.176
2009	22	44	0.099	0.28	0.548
2009	22	45	0.150	0.00	0.043
2009	22	46	-0.008	0.00	0.001
2009	22	47	0.053	0.00	0.003
2009	22	48	0.107	0.00	0.000
2009	22	49	-0.102	0.03	0.299
2009	22	50	0.126	0.00	0.251
2009	22	51	0.216	0.00	0.050

2009	22	52	-0.052	0.00	0.043
2009	22	53	-0.061	0.00	0.024
2009	22	54	0.137	0.00	0.061
2009	23	26	-0.063	0.84	0.940
2009	23	28	0.003	0.00	0.001
2009	23	29	-0.092	0.00	0.000
2009	23	30	-0.113	0.00	0.000
2009	23	32	0.199	0.00	0.004
2009	23	33	0.025	0.00	0.000
2009	23	34	-0.170	0.00	0.003
2009	23	36	-0.250	0.00	0.276
2009	23	37	-0.252	0.00	0.322
2009	23	38	-0.108	0.00	0.353
2009	23	39	0.026	0.00	0.001
2009	23	40	-0.034	0.00	0.000
2009	23	41	0.118	0.15	0.690
2009	23	42	-0.042	0.00	0.000
2009	23	43	0.048	0.00	0.000
2009	23	44	-0.064	0.00	0.000
2009	23	45	0.048	0.00	0.000
2009	23	46	-0.134	0.00	0.035
2009	23	47	-0.036	0.00	0.034
2009	23	48	0.016	0.00	0.041
2009	23	49	0.019	0.00	0.000
2009	23	50	0.178	0.00	0.000
2009	23	51	0.148	0.00	0.000
2009	23	52	-0.158	0.00	0.000
2009	23	53	-0.078	0.00	0.000
2009	23	54	-0.129	0.00	0.000
2009	26	28	-0.096	0.00	0.001
2009	26	29	-0.114	0.00	0.000
2009	26	30	-0.224	0.00	0.000
2009	26	32	0.054	0.00	0.004
2009	26	33	0.022	0.00	0.000
2009	26	34	-0.190	0.00	0.003
2009	26	36	-0.283	0.00	0.273
2009	26	37	0.071	0.00	0.315
2009	26	38	-0.054	0.00	0.347
2009	26	39	-0.105	0.00	0.000
2009	26	40	0.171	0.00	0.000
2009	26	41	-0.167	0.14	0.679
2009	26	42	-0.128	0.00	0.000
2009	26	43	-0.229	0.00	0.000
2009	26	44	0.247	0.00	0.000
2009	26	45	-0.292	0.00	0.000
2009	26	46	-0.224	0.00	0.033

2009	26	47	-0.090	0.00	0.033
2009	26	48	-0.246	0.00	0.039
2009	26	49	0.162	0.00	0.000
2009	26	50	-0.098	0.00	0.000
2009	26	51	0.219	0.00	0.000
2009	26	52	0.231	0.00	0.000
2009	26	53	-0.216	0.00	0.000
2009	26	54	0.037	0.00	0.000
2009	28	29	0.605	0.40	0.528
2009	28	30	0.117	0.67	0.790
2009	28	32	0.444	0.00	0.170
2009	28	33	-0.054	0.00	0.002
2009	28	34	-0.049	0.19	0.565
2009	28	36	-0.063	0.00	0.015
2009	28	37	0.027	0.00	0.014
2009	28	38	-0.209	0.00	0.019
2009	28	39	0.013	0.07	0.442
2009	28	40	-0.192	0.13	0.593
2009	28	41	0.171	0.00	0.032
2009	28	42	0.073	0.15	0.665
2009	28	43	-0.033	0.00	0.042
2009	28	44	0.063	0.00	0.007
2009	28	45	0.525	0.81	0.935
2009	28	46	-0.103	0.00	0.066
2009	28	47	-0.134	0.00	0.082
2009	28	48	0.161	0.00	0.050
2009	28	49	0.328	0.00	0.070
2009	28	50	0.135	0.00	0.064
2009	28	51	0.044	0.00	0.000
2009	28	52	-0.298	0.00	0.000
2009	28	53	-0.049	0.00	0.000
2009	28	54	0.067	0.03	0.033
2009	29	30	0.218	0.32	0.508
2009	29	32	0.323	0.00	0.311
2009	29	33	-0.204	0.00	0.032
2009	29	34	0.056	0.16	0.504
2009	29	36	-0.132	0.00	0.009
2009	29	37	-0.057	0.00	0.008
2009	29	38	-0.249	0.00	0.014
2009	29	39	-0.014	0.11	0.353
2009	29	40	-0.214	0.08	0.687
2009	29	41	0.023	0.00	0.031
2009	29	42	0.116	0.07	0.663
2009	29	43	-0.172	0.00	0.080
2009	29	44	-0.098	0.00	0.041
2009	29	45	0.333	0.39	0.520

2009	29	46	-0.061	0.00	0.137
2009	29	47	-0.217	0.00	0.172
2009	29	48	0.022	0.00	0.104
2009	29	49	0.381	0.03	0.168
2009	29	50	0.183	0.02	0.156
2009	29	51	-0.159	0.00	0.016
2009	29	52	-0.442	0.00	0.013
2009	29	53	-0.071	0.00	0.005
2009	29	54	-0.107	0.00	0.047
2009	30	32	-0.098	0.00	0.126
2009	30	33	0.190	0.00	0.005
2009	30	34	0.244	0.20	0.460
2009	30	36	-0.056	0.00	0.004
2009	30	37	-0.078	0.00	0.004
2009	30	38	0.075	0.00	0.008
2009	30	39	0.390	0.06	0.504
2009	30	40	-0.090	0.24	0.595
2009	30	41	0.036	0.00	0.031
2009	30	42	0.156	0.12	0.567
2009	30	43	-0.156	0.00	0.064
2009	30	44	-0.237	0.00	0.017
2009	30	45	0.252	0.66	0.797
2009	30	46	-0.110	0.00	0.040
2009	30	47	0.051	0.00	0.052
2009	30	48	-0.138	0.00	0.028
2009	30	49	-0.084	0.00	0.061
2009	30	50	0.299	0.00	0.055
2009	30	51	-0.144	0.00	0.001
2009	30	52	-0.041	0.00	0.001
2009	30	53	-0.154	0.00	0.000
2009	30	54	0.242	0.03	0.034
2009	32	33	-0.020	0.00	0.000
2009	32	34	-0.119	0.04	0.230
2009	32	36	-0.013	0.00	0.071
2009	32	37	0.194	0.00	0.073
2009	32	38	-0.146	0.00	0.077
2009	32	39	0.191	0.00	0.093
2009	32	40	0.139	0.04	0.230
2009	32	41	-0.047	0.00	0.028
2009	32	42	0.110	0.02	0.286
2009	32	43	-0.136	0.00	0.003
2009	32	44	0.287	0.00	0.000
2009	32	45	0.296	0.00	0.147
2009	32	46	0.201	0.05	0.400
2009	32	47	0.300	0.06	0.579
2009	32	48	0.463	0.06	0.333

2009	32	49	0.178	0.00	0.080
2009	32	50	0.203	0.00	0.078
2009	32	51	0.250	0.00	0.000
2009	32	52	0.077	0.00	0.000
2009	32	53	0.186	0.00	0.000
2009	32	54	0.115	0.00	0.007
2009	33	34	0.060	0.00	0.001
2009	33	36	-0.214	0.00	0.000
2009	33	37	0.065	0.00	0.000
2009	33	38	-0.151	0.00	0.000
2009	33	39	0.133	0.00	0.010
2009	33	40	0.171	0.00	0.026
2009	33	41	-0.090	0.00	0.000
2009	33	42	-0.114	0.00	0.001
2009	33	43	0.282	0.00	0.137
2009	33	44	-0.015	0.32	0.349
2009	33	45	0.017	0.00	0.001
2009	33	46	-0.220	0.00	0.000
2009	33	47	0.168	0.00	0.000
2009	33	48	-0.072	0.00	0.000
2009	33	49	-0.182	0.02	0.124
2009	33	50	-0.140	0.02	0.094
2009	33	51	0.166	0.02	0.397
2009	33	52	0.265	0.02	0.397
2009	33	53	-0.016	0.00	0.332
2009	33	54	0.145	0.02	0.397
2009	34	36	0.328	0.00	0.057
2009	34	37	0.245	0.00	0.062
2009	34	38	0.144	0.00	0.063
2009	34	39	0.079	0.00	0.283
2009	34	40	-0.121	0.14	0.501
2009	34	41	-0.197	0.00	0.038
2009	34	42	-0.270	0.19	0.699
2009	34	43	-0.095	0.00	0.026
2009	34	44	-0.001	0.00	0.002
2009	34	45	0.140	0.21	0.560
2009	34	46	0.170	0.00	0.085
2009	34	47	0.053	0.00	0.104
2009	34	48	-0.186	0.00	0.082
2009	34	49	-0.233	0.00	0.030
2009	34	50	0.083	0.00	0.027
2009	34	51	-0.117	0.00	0.000
2009	34	52	-0.032	0.00	0.000
2009	34	53	0.117	0.00	0.000
2009	34	54	0.153	0.00	0.033
2009	36	37	0.198	0.67	0.869

2009	36	38	0.294	0.49	0.845
2009	36	39	0.127	0.00	0.019
2009	36	40	-0.109	0.00	0.005
2009	36	41	-0.129	0.00	0.309
2009	36	42	-0.083	0.00	0.008
2009	36	43	-0.269	0.00	0.000
2009	36	44	0.284	0.00	0.000
2009	36	45	-0.081	0.00	0.004
2009	36	46	-0.028	0.00	0.243
2009	36	47	-0.015	0.00	0.248
2009	36	48	-0.059	0.00	0.244
2009	36	49	-0.146	0.00	0.000
2009	36	50	0.022	0.00	0.000
2009	36	51	-0.105	0.00	0.000
2009	36	52	0.140	0.00	0.000
2009	36	53	0.183	0.00	0.000
2009	36	54	0.122	0.00	0.000
2009	37	38	-0.065	0.53	0.880
2009	37	39	0.067	0.00	0.022
2009	37	40	0.047	0.00	0.004
2009	37	41	-0.379	0.05	0.347
2009	37	42	-0.448	0.00	0.009
2009	37	43	-0.131	0.00	0.000
2009	37	44	-0.072	0.00	0.000
2009	37	45	0.043	0.00	0.003
2009	37	46	0.018	0.03	0.267
2009	37	47	0.095	0.00	0.269
2009	37	48	-0.307	0.04	0.265
2009	37	49	-0.179	0.00	0.000
2009	37	50	-0.136	0.00	0.000
2009	37	51	0.036	0.00	0.000
2009	37	52	-0.022	0.00	0.000
2009	37	53	-0.105	0.00	0.000
2009	37	54	0.170	0.00	0.000
2009	38	39	-0.237	0.00	0.015
2009	38	40	-0.224	0.00	0.008
2009	38	41	-0.039	0.05	0.419
2009	38	42	-0.211	0.00	0.013
2009	38	43	-0.386	0.00	0.000
2009	38	44	0.053	0.00	0.000
2009	38	45	-0.260	0.00	0.007
2009	38	46	0.009	0.03	0.213
2009	38	47	-0.158	0.00	0.221
2009	38	48	-0.105	0.02	0.215
2009	38	49	-0.225	0.00	0.000
2009	38	50	-0.232	0.00	0.000

2009	38	51	-0.150	0.00	0.000
2009	38	52	0.038	0.00	0.000
2009	38	53	-0.202	0.00	0.000
2009	38	54	0.151	0.00	0.000
2009	39	40	0.340	0.00	0.324
2009	39	41	-0.024	0.04	0.031
2009	39	42	0.007	0.00	0.381
2009	39	43	-0.061	0.00	0.059
2009	39	44	0.001	0.00	0.032
2009	39	45	-0.104	0.06	0.439
2009	39	46	-0.093	0.00	0.105
2009	39	47	0.271	0.06	0.114
2009	39	48	-0.078	0.03	0.106
2009	39	49	0.051	0.00	0.084
2009	39	50	0.156	0.00	0.082
2009	39	51	0.007	0.00	0.000
2009	39	52	0.101	0.00	0.000
2009	39	53	-0.206	0.00	0.000
2009	39	54	0.108	0.00	0.033
2009	40	41	-0.106	0.00	0.031
2009	40	42	-0.178	0.28	0.670
2009	40	43	0.021	0.00	0.057
2009	40	44	0.048	0.00	0.033
2009	40	45	-0.359	0.09	0.615
2009	40	46	0.040	0.00	0.119
2009	40	47	0.439	0.00	0.140
2009	40	48	0.044	0.00	0.098
2009	40	49	0.091	0.00	0.144
2009	40	50	0.070	0.00	0.133
2009	40	51	0.437	0.00	0.001
2009	40	52	0.378	0.00	0.001
2009	40	53	-0.149	0.00	0.000
2009	40	54	0.234	0.00	0.034
2009	41	42	0.174	0.00	0.032
2009	41	43	-0.101	0.00	0.009
2009	41	44	-0.181	0.00	0.001
2009	41	45	-0.126	0.00	0.031
2009	41	46	-0.152	0.00	0.035
2009	41	47	-0.293	0.00	0.047
2009	41	48	0.379	0.00	0.034
2009	41	49	0.081	0.00	0.024
2009	41	50	0.103	0.00	0.022
2009	41	51	-0.171	0.00	0.000
2009	41	52	-0.202	0.00	0.000
2009	41	53	-0.100	0.00	0.000
2009	41	54	-0.217	0.00	0.009

2009	42	43	0.088	0.03	0.038
2009	42	44	-0.128	0.00	0.005
2009	42	45	-0.027	0.10	0.658
2009	42	46	-0.219	0.00	0.156
2009	42	47	-0.064	0.00	0.182
2009	42	48	0.289	0.00	0.122
2009	42	49	0.164	0.02	0.144
2009	42	50	0.089	0.02	0.136
2009	42	51	-0.232	0.00	0.000
2009	42	52	-0.132	0.00	0.000
2009	42	53	0.177	0.00	0.000
2009	42	54	-0.206	0.00	0.033
2009	43	44	-0.201	0.09	0.170
2009	43	45	0.187	0.00	0.036
2009	43	46	-0.131	0.00	0.001
2009	43	47	-0.036	0.00	0.002
2009	43	48	-0.041	0.00	0.000
2009	43	49	-0.168	0.00	0.039
2009	43	50	-0.245	0.00	0.033
2009	43	51	-0.145	0.00	0.044
2009	43	52	-0.151	0.00	0.035
2009	43	53	-0.042	0.00	0.013
2009	43	54	-0.149	0.00	0.054
2009	44	45	-0.045	0.00	0.005
2009	44	46	-0.109	0.00	0.000
2009	44	47	-0.082	0.00	0.000
2009	44	48	0.168	0.00	0.000
2009	44	49	0.097	0.00	0.118
2009	44	50	-0.102	0.00	0.103
2009	44	51	0.308	0.00	0.058
2009	44	52	0.227	0.00	0.045
2009	44	53	0.046	0.00	0.021
2009	44	54	0.106	0.00	0.054
2009	45	46	0.119	0.00	0.051
2009	45	47	-0.072	0.00	0.065
2009	45	48	-0.002	0.00	0.036
2009	45	49	-0.004	0.00	0.060
2009	45	50	0.138	0.00	0.055
2009	45	51	0.069	0.00	0.000
2009	45	52	-0.527	0.00	0.000
2009	45	53	0.065	0.00	0.000
2009	45	54	-0.027	0.01	0.033
2009	46	47	0.524	0.71	0.760
2009	46	48	0.335	0.61	0.740
2009	46	49	0.085	0.00	0.074
2009	46	50	0.122	0.00	0.074

2009	46	51	0.095	0.00	0.000
2009	46	52	-0.132	0.00	0.000
2009	46	53	0.253	0.00	0.000
2009	46	54	-0.167	0.00	0.001
2009	47	48	0.211	0.49	0.674
2009	47	49	-0.071	0.00	0.103
2009	47	50	0.172	0.00	0.102
2009	47	51	0.098	0.00	0.000
2009	47	52	0.323	0.00	0.000
2009	47	53	0.137	0.00	0.000
2009	47	54	0.115	0.00	0.002
2009	48	49	0.197	0.00	0.054
2009	48	50	0.069	0.00	0.054
2009	48	51	0.095	0.00	0.000
2009	48	52	0.156	0.00	0.000
2009	48	53	0.249	0.00	0.000
2009	48	54	-0.145	0.00	0.000
2009	49	50	0.409	0.80	0.883
2009	49	51	-0.074	0.00	0.008
2009	49	52	-0.043	0.00	0.007
2009	49	53	0.347	0.00	0.004
2009	49	54	-0.074	0.00	0.014
2009	50	51	-0.195	0.00	0.002
2009	50	52	0.043	0.00	0.002
2009	50	53	0.375	0.00	0.001
2009	50	54	0.251	0.00	0.008
2009	51	52	0.022	0.81	0.900
2009	51	53	-0.144	0.25	0.763
2009	51	54	0.121	0.81	0.929
2009	52	53	0.145	0.32	0.835
2009	52	54	0.389	0.77	0.923
2009	53	54	0.034	0.20	0.786
